# Dispersal changes soil bacterial interactions with fungal wood decomposition

**DOI:** 10.1038/s43705-023-00253-5

**Published:** 2023-05-03

**Authors:** Cong Wang, Gabriel Reuben Smith, Cheng Gao, Kabir G. Peay

**Affiliations:** 1 State Key Laboratory of Mycology, Institute of Microbiology, Chinese Academy of Sciences, 100101 Beijing, China; 2 Key Laboratory of Vegetation Restoration and Management of Degraded Ecosystems and Guangdong Provincial Key Laboratory of Applied Botany, South China Botanical Garden, Chinese Academy of Sciences, Guangzhou, 510650 China; 3 Global Ecosystem Ecology, Department of Environmental Systems Science, Institute of Integrative Biology, ETH Zürich, Zürich, 8092 Switzerland; 4 Department of Biology, Stanford University, Stanford, CA 94305 USA; 5 Department of Earth System Science, Stanford University, Stanford, CA 94305 USA

**Keywords:** Microbial ecology, Microbial ecology, Biogeochemistry

## Abstract

Although microbes are the major agent of wood decomposition - a key component of the carbon cycle - the degree to which microbial community dynamics affect this process is unclear. One key knowledge gap is the extent to which stochastic variation in community assembly, e.g. due to historical contingency, can substantively affect decomposition rates. To close this knowledge gap, we manipulated the pool of microbes dispersing into laboratory microcosms using rainwater sampled across a transition zone between two vegetation types with distinct microbial communities. Because the laboratory microcosms were initially identical this allowed us to isolate the effect of changing microbial dispersal directly on community structure, biogeochemical cycles and wood decomposition. Dispersal significantly affected soil fungal and bacterial community composition and diversity, resulting in distinct patterns of soil nitrogen reduction and wood mass loss. Correlation analysis showed that the relationship among soil fungal and bacterial community, soil nitrogen reduction and wood mass loss were tightly connected. These results give empirical support to the notion that dispersal can structure the soil microbial community and through it ecosystem functions. Future biogeochemical models including the links between soil microbial community and wood decomposition may improve their precision in predicting wood decomposition.

## Introduction

Dead wood holds about 20% of the carbon (C) (~72 Pg C) in forest vegetation [1], a proportion that may increase as intense climate extremes and disturbances exacerbate tree mortality [2–4]. The decay of wood releases nutrients and C, influencing plant growth and our planet’s C cycle [5, 6]. Understanding the factors determining wood decomposition rates are thus essential for building accurate ecosystem and climate models. Previous studies have quantified the effects of climate factors and wood traits on wood decomposition [7]. However, though our understanding of the major role played by microbes in wood decomposition is growing [8–10], the extent to which fine-scale differences in microbial community assembly should translate to patterns in wood decomposition remains unclear.

Although microbes are key agents of decomposition in general, variation in their activity rates has historically been considered largely a proxy of climate factors since microbial activity can be constrained by temperature and moisture [11]. However, for dead wood, wood nitrogen (N) content and debris diameter are both better predictors than climate factors at a global scale [7], factors which may interact with microbial communities at a finer spatial scale. Indeed, fungal colonization is itself a superior predictor of wood decomposition than climate factors at regional scales [12]. Unlike climate or wood diameter, local availability of nitrogen is itself determined by the microbial community and must be imported by wood-decay fungi from underlying soil to subsidize their decomposition activity [13–15]. These results suggest that it is important to identify local-scale controls on microbial community composition for precise estimate of wood decomposition [16].

Specifically, arrival order and priority effects appear to massively influence community development and wood decomposition. Despite this, few studies investigate how natural, landscape-scale variation in dispersal and community assembly of wood-decay fungi might constrain this important ecosystem process [16]. It is also not yet clear how such patterns might interact with spatial turnover in soil microbial communities, which are themselves also potentially shaped by dispersal limitation [17–20].

Additionally, current opinion on microbial wood decomposition is that fungi are the major players while total contribution of bacteria to wood decomposition appears to be minor [21]. For example, Hu, Yesilonis [22] investigated fungal and bacterial composition during wood decomposition in six temperate upland forests, and found that only fungal community composition significantly correlated with wood mass loss. However, dead wood is very nitrogen-scarce, and wood inhabiting bacteria have been shown to promote fungal wood decomposition through N fixation [23, 24]. Moreover, fungi in wood mine soil nutrients through hyphae to support their growth and activity [13–15]. Therefore, soil bacteria may indirectly participate in wood decomposition through their key roles in soil nitrogen cycling [25]. On the contrary, substrate release and fungal biomass formation during wood decay may affect soil bacterial community through substrate acidification, fungal metabolites or degradable C source, etc. [26–28]. However, there is little documentation on the interactions between soil bacterial community and wood decomposition [29].

Here, we investigate how dispersal-driven patterns in soil microbial community assembly affect soil biogeochemical cycling and wood decomposition. We used a distance gradient consisting of seventeen sites, ranging from near to far from the edge of pine forests at Point Reyes National Seashore in California, United States (Supplementary Fig. S1). Aerially dispersed microbial propagules sourced from the forest occur at high concentrations near the edge, but decrease in abundance as distance increases [30]. Rain water containing propagules at each site was collected every 48 days [16], and was used to inoculate wood-soil microcosms incubated under controlled laboratory conditions. This allowed us to disentangle the effects of microbial community assembly on ecosystem function from those of abiotic environmental variation. After a year of incubation, we measured soil microbial community characteristics (fungal and bacterial diversity and community composition) and ecosystem functioning (wood decomposition and shifts of soil carbon and nitrogen) across the distance gradient. We hypothesized that the soil microbial community would show significant turnover across the distance gradient, as previously seen with fungal communities in wood blocks [16]. We anticipated, moreover, that microbial community turnover would give rise to variation in soil biogeochemical cycling and wood decomposition, in which soil bacteria would also participate in wood decomposition like fungi by affecting the nutrient supply of fungi.

## Results

### Soil fungal and bacterial community characteristics across the distance gradient

**Fig. 1:**
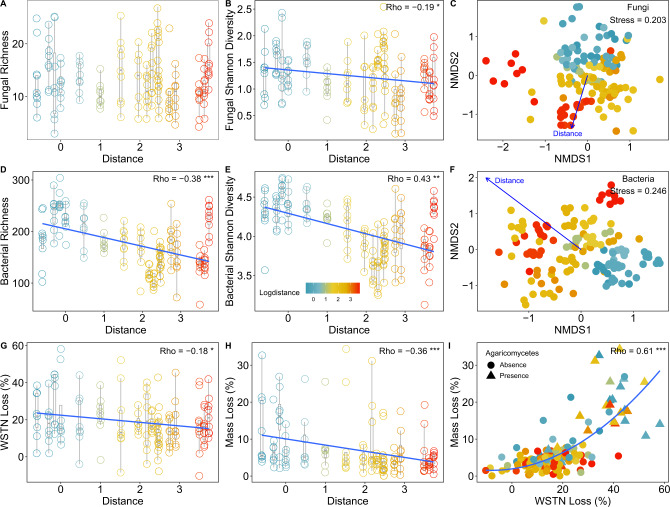
Soil microbial community and ecosystem functions across the distance gradient from the forest edge. **A**, **B** Soil fungal Shannon diversity but not richness decreased as distance increased from the forest edge. **C** Visualization of soil fungal community composition across the distance gradient. **D**, **E** Soil bacterial alpha diversity decreased as distance increased from the forest edge. **F** Visualization of soil bacterial community composition across the distance gradient. **G**, **H** Both wood mass loss and soil WSTN reduction decreased with increased distance from the forest edge. **I** Relationships between percent wood mass loss and percent soil WSTN reduction. *n* = 142 for all analysis. *P < 0.05, **P < 0.01, ***P < 0.001.

**Fig. 2:**
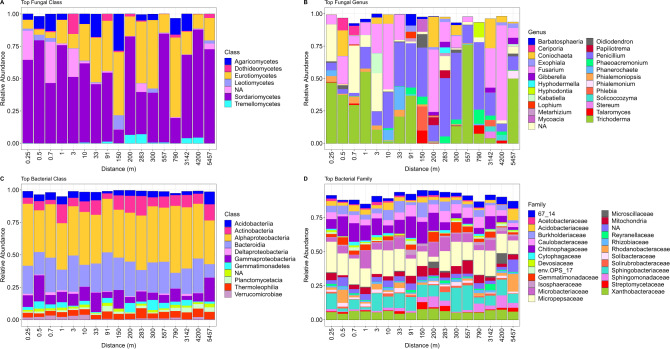
The relative abundance of soil fungi and bacteria across the distance gradient (Distance from the pine forest edge). **A**, **B** The relative abundance of top fungal classes and genera. **C**, **D** The relative abundance of top bacterial classes and families.

Soil fungal community composition significantly changed (envfit analysis: R^2^ = 0.486, *P* < 0.001) accompanying by decrease in fungal Shannon diversity as distance increased from the forest edge (Fig. 1B, C). The dominant fungal phyla were Ascomycota and Basidiomycota, and the top fungal classes were Sordariomycetes, Eurotiomycetes, Agaricomycetes, Dothideomycetes, Leotiomycetes and Tremellomycetes (Fig. 2A and Supplementary Fig. S2A).

Bacterial richness and Shannon diversity both decreased as distance increased from the forest edge (*P* < 0.05, Fig. 1D, E), and bacterial community composition also changed significantly across the distance gradient (envfit analysis: R^2^ = 0.591, *P* < 0.001, Fig. 1F). The dominant bacterial phyla were *Proteobacteria*, *Bacteroidetes*, *Actinobacteria*, *Acidobacteria*, *Gemmatimonadetes* and *Planctomycetes* (Supplementary Fig. S2B).

### Ecosystem functions across the distance gradient

Microbial inoculation decreased soil total C, total N, water-soluble organic C (WSOC), and water-soluble total N (WSTN) compared to controls treated only with ddH_2_O (Supplementary Fig. S4). As the easily accessible form of organic matter to microbes, shift of soil WSOC and WSTN (avg: 52% and 19%, respectively) were larger than shift of soil total C and N (avg: 10% and 5%, respectively). However, only percent WSTN reduction showed a strong spatial pattern (ρ = −0.18, *P* < 0.05, Fig. 1G, Supplementary Fig. S5) similar to that of percent mass loss of wood (ρ = −0.36, *P* < 0.001, Fig. 1H, Supplementary Fig. S5). Accordingly, soil WSTN reduction and wood mass loss were significantly correlated (ρ = 0.61, *P* < 0.001, Fig. 1I, Supplementary Fig. S5).

### Relationships between soil microbial community characteristics and WSTN and wood mass loss

**Fig. 3:**
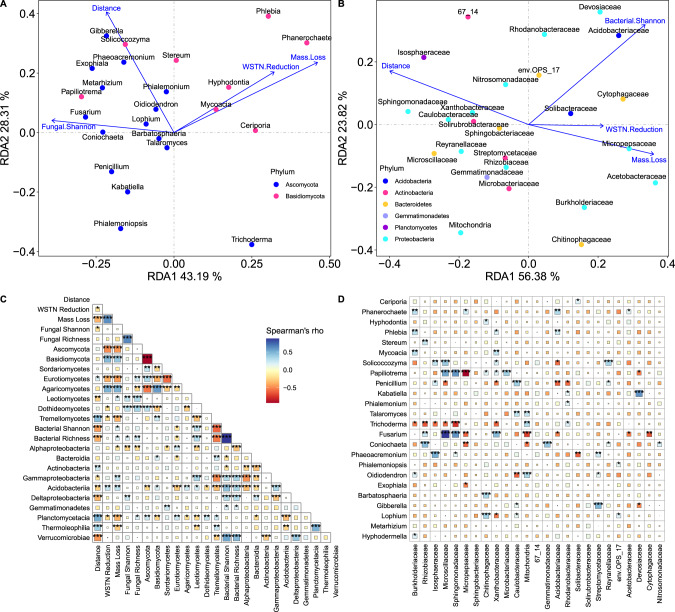
Relationships between soil microbial community characteristics, wood mass loss percent and soil WSTN reduction percent. **A** Redundancy analysis (RDA) ordination biplot of the relative abundance of top fungal genera and wood mass loss, soil WSTN reduction, fungal Shannon diversity across the distance gradient from the edge of forest. **B** RDA ordination biplot of the relative abundance of top bacterial family and wood mass loss, soil WSTN reduction, bacterial Shannon diversity across the distance gradient from the edge of forest. **C** Correlations among soil microbial alpha diversity, relative abundance, wood mass loss and soil WSTN reduction across the distance gradient from the edge of forest. **D** Correlations between top fungal genera and top bacterial family. WSTN water-soluble total nitrogen. *n* = 142 for all analysis. *P < 0.05, **P < 0.01, ***P < 0.001.

Soil fungal community richness and Shannon diversity were not correlated with wood mass loss or soil WSTN reduction (Fig. 3C). As for specific soil fungal taxa, the phylum Ascomycota negatively correlated with wood mass loss and soil WSTN reduction, while the phylum Basidiomycota positively correlated with them (Fig. 3C). Redundancy analysis (RDA) showed that soil fungal community composition at genus level significantly correlated with wood mass loss and soil WSTN reduction (R^2^ = 0.022, *P* = 0.002 and R^2^ = 0.013, *P* = 0.001, respectively, Fig. 3A, Supplementary Fig. S6A), and RDA further showed that genera of Ascomycota mainly negative correlated with wood mass loss and soil WSTN reduction while genera of Basidiomycota mainly positively correlated with them (Fig. 3A, Supplementary Fig. S6A).

The soil bacterial community also had multiple links with wood mass loss and soil WSTN loss. Bacterial taxonomic richness and Shannon diversity positively correlated with wood mass loss (Fig. 3C). RDA showed that soil bacterial community composition at family level significantly correlated with wood mass loss and soil WSTN reduction (R^2^ = 0.041, *P* = 0.001 and R^2^ = 0.016, *P* = 0.001, respectively, Fig. 3B, Supplementary Fig. S6B). In detail, *Chitinophagaceae*, *Burkholderiaceae*, *Acidobacteriaceae*, *Rhodanobacteraceae*, *Acetobacteraceae*, and *Cytophagaceae* positively correlated with wood mass loss or soil WSTN reduction while *Caulobacteraceae*, *Sphingomonadaceae*, *Isosphaeraceae*, *Solirubrobacteraceae*, *Microscillaceae*, *Reyranellaceae* negatively correlated with them (Fig. 3B, Supplementary Fig. S6B).

**Fig. 4:**
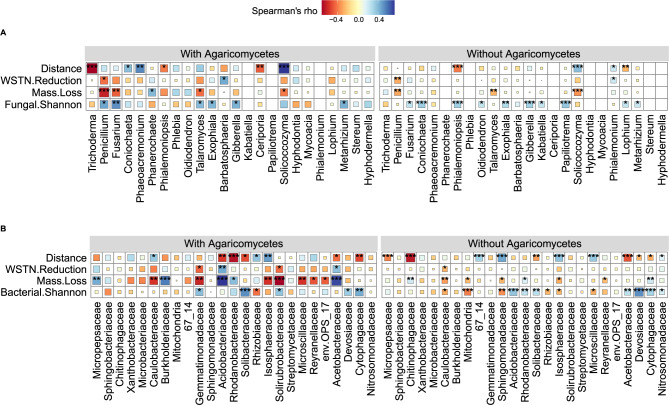
Effect of the presence and absence of Agaricomycetes on the relationships among soil microbes, wood mass loss percent and soil WSTN reduction percent. **A** Relationships between top fungal genera and wood mass loss, soil WSTN reduction, fungal Shannon diversity across the distance gradient from the edge of forest. **B** Relationships between top bacterial families and wood mass loss, soil WSTN reduction, bacterial Shannon diversity across the distance gradient from the edge of forest. With Agaricomycetes: *n* = 31, without Agaricomycetes: *n* = 111, WSTN: soil water-soluble total *N*. *P < 0.05, **P < 0.01, ***P < 0.001.

The relative abundance of Agaricomycetes was significantly correlated with wood mass loss (Fig. 3C), and RDA showed that the top fungal genera significantly correlating with wood mass loss belonged to Agaricomycetes (Fig. 3A). However, not all samples contained Agaricomycetes (Fig. 2A). To analyze the effects of Agaricomycetes, we grouped samples by with and without Agaricomycetes, and further analyzed the relationships between soil microbes and wood mass loss, soil WSTN reduction. The results showed that the correlations between soil bacteria and wood mass loss, soil WSTN reduction were stronger in samples with Agaricomycetes (Fig. 4B).

## Discussion

Prior work shows that microbial community turnover influences decomposition rate [31, 32], but identifying the functional impact of natural, in situ variation in microbial communities remains challenging. Recreating a natural microbial dispersal gradient under controlled laboratory settings, we observed distinct spatial patterns in community assembly (Figs. 1 and 2). These dispersal-driven patterns affected soil WSTN reduction and wood decomposition (Fig. 1). We thus find a role for natural dispersal limitation of both fungi and bacteria in shaping these important ecosystem processes. The role of dispersal limitation in fungal community assembly is widely recognized, while its role in structuring bacterial community is still inconclusive [33, 34]. Bell [35] excluded environmental cofounding factors by using a reciprocal transplant experiment, through which Bell measured bacterial dispersal in a woodland, and found that the role of dispersal limitation is minor. This result is in contrast with the distinct bacterial spatial pattern in our study (Fig. 1F). The inconsistency may be attributed to different environment and range of distance and time. Bell’s study within a woodland measured bacterial dispersal at a 0–500 m distance gradient in 28 days, while our study identified bacterial dispersal across a half year at a 0–5000 m distance gradient from the edge of forest.

As expected, we observed multiple links between soil fungal communities and wood decomposition. As in prior work [16], the relative abundance of Agaricomycetes (*Phanerochaete*, *Phlebia*, etc.) was positively correlated with wood mass loss (Fig. 3A, C). This is reasonable, as the Agaricomycetes contains many well-known wood-decay fungi [36], and species in underlying soil may be physically linked to mycelia that are actively decomposing woody debris above [37, 38]. Indeed, the shared fungal ASVs (amplicon sequence variants) accounted for ca. 60% of wood fungal ASVs and 55% of soil fungal ASVs (Supplementary Fig. S3), which is in accordance with a previous study in field situation [38]. Furthermore, at the phylum level, the phylum Ascomycota had a negative relationship with wood mass loss while the phylum Basidiomycota had a positive relationship with wood mass loss (Fig. 3C). This result can be supported by previous finding that Basidiomycota hold genes for lignin degradation while Ascomycota hold genes for cellulose degradation in the context of wood [24]. However, though previous studies found that fungal richness in woody debris was negatively correlated with mass loss [31], the connections between taxonomic richness or diversity of soil fungi and wood decomposition were insignificant (Fig. 3C). This is likely because negative diversity-function relationships in wood-decay fungi are driven by competition for space in the actual dead wood substrate [39], and such dynamics may not translate to the underlying soil community. We thus find that some, but not all, patterns linking fungal communities to wood decomposition are symmetrical across woody debris and underlying soil.

As anticipated, soil bacteria community was also correlated with wood mass loss. The connections between bacteria community and wood mass loss can be attributed to two pathways. One of the pathways is that soil bacteria affect woody decomposition through modifying soil nutrients for fungal activity, as deadwood is very nitrogen-scarce and fungi on wood translocate nitrogen and nutrient from soil to support their decomposition [13–15]. Indeed, wood mass loss was tightly correlated with soil WSTN reduction in our study (Fig. 1G). Furthermore, some bacterial taxa with N-fixing ability were significantly correlated with soil WSTN reduction and wood mass loss (Fig. 3B, Supplementary Fig. S6B). *Isosphaeraceae*, *Microscillaceae*, *Sphingomonadaceae*, *Burkholderiaceae* and *Rhizobiaceae* have N-fixing genes [24, 40, 41]. Interestingly, among these N-fixing taxa, *Burkholderiaceae* showed significant positive correlations with wood mass loss and fungal genera of Agaricomycetes (*Phanerochaete*, *Phlebia* and *Mycoacia*, Fig. 3A, D), which is consistent with previous report that *Burkholderiaceae* was pervasively coupled with wood decay fungi [42]. In contrast, *Isophaeraceae* and *Microscillaceae* were negatively correlated with soil WSTN reduction and wood mass loss (Fig. 3B, Supplementary Fig. S6B). The negative correlations between these N-fixing taxa and wood mass loss are in contrast with previous report that the presence of N-fixing bacteria facilitate fungal wood decomposition [23, 24]. Such negative correlations may be driven by dispersal or changes in microhabitat with wood decay.

Another pathway linking soil bacteria and wood mass is that substrate release and fungal activity may affect soil bacteria during wood decomposition. Previous studies found that substrate acidification, decrease in C: N ratio, release of fungal metabolites and formation of fungal biomass with advanced wood decomposition can drive succession of wood inhabiting bacteria [26, 27]. In our study, several acidophilic bacterial taxa, i.e., *Micropepsaceae* [43], *Acidobacteriaceae* and *Acetobacteraceae* [44], were positively correlated wood mass loss (Fig. 3B, Supplementary Fig. S6B). *Chitinophagaceae*, a chitinolytic taxon [45], was also positively correlated with wood mass loss (Fig. 3B, Supplementary Fig. S6B). Shifts of these taxa is likely correlated with substrate acidification and fungal biomass accumulation during wood decomposition, indicating that microhabitat changes due to wood decay may also affect soil bacteria. However, in contrast to the decrease in C: N ratio of wood part [26, 27], soil N content reduced and soil C: N ratio increased during wood decomposition (Fig. 3C, Supplementary Fig. S5). This is a different change between wood part and soil part during wood decomposition, and such difference may also occur in other nutrients due to fungi translocate them from soil to wood [38]. Such distinct changes between wood and soil part may cause different shifts of soil bacteria community of these two parts, which deserve further investigation. In this study, decrease in soil C: N ratio seems to mainly affect *Xanthobacteraceae*, *Solibacteraceae*, *Rhizobiaceae* and *Solirubrobacteraceae* (Supplementary Fig. S5). However, there is little know about whether the different shift of C: N ratio between wood and soil during wood decomposition would cause divergent bacteria community.

Correlations between soil bacteria and wood mass loss, soil WSTN reduction were strengthened by the presence of Agaricomycetes (Fig. 4B). This can be attributed to more advanced wood decay in samples with Agaricomycetes (Fig. 1I). Previous study in situ found that nutrients translocation between soil and wood inhabiting fungi were more active during late decay stage than that in early decay stage [38]. The more intensive interactions between soil and wood inhabiting fungi may therefore tighten correlations between soil bacteria and wood mass loss, soil WSTN reduction.

### Summary

As we hypothesized, results of this study showed that dispersal limitation could affect fungal and bacterial community assembly, and then affect ecosystem functions. This constitutes a valuable quantitative empirical example, supporting the fundamental ecological notion that dispersal can shape local microbial community assembly and through it ecosystem functions. Furthermore, another novelty aspect of our study is that results of this study demonstrated quantitative correlations between aboveground wood decomposition and belowground soil microbial community and ecosystem function. Such connections should be pervasive in the field and may exert large effects on ecosystem functions, as about half of deadwood is in contact with soil in situ [46, 47] and on these deadwood fungi actively translocate nutrients from soil [38]. Future study untangling relationships among wood inhabiting microbes, soil microbes, wood decomposition and soil ecosystem processes could be help for more precise predictions of wood decomposition in a changing climate.

## Materials and methods

### Study site

Our study was conducted at Point Reyes National Seashore, located in Marin County, California (38°04′N, 122°50′W). This study area has typical Mediterranean climate with cool, wet winters and hot, dry summers. The mean annual rainfall is about 43 cm, with most of the rainfall distribution in the winter months (November to February). The mean annual temperature of this area is 11 °C, and the average temperate in January and September is 10 °C and 13.5 °C, respectively. The vegetation at Point Reyes National Seashore is a heterogenous mosaic of forest, grass, and scrub land (Supplementary Fig. S1). Mono-dominant coastal forests here are composed of *Pinus muricata D. Don*, and the grass and scrub land mainly cover with *Baccharis pilularis*, *Toxicodendron diversiloba* and *Rubus ursinus*.

### Experiment design

To evaluate the effect of dispersal on soil microbial community and ecosystem functions, we collected rainwater along a distance gradient from the forest edge, then used this rainwater to inoculate laboratory wood-soil microcosms. The distance gradient had seventeen sites ranging in distance from *P. muricata* forests from directly adjacent (0.25 m) to ~5.5 km away (Supplementary Fig. S1; [16, 48]). Movement through airborne spores and other propagules is a major means of fungal and bacterial dispersal, and rainfall can create large amounts of bioparticles with high diversity of fungi and bacteria [49]. With our laboratory approach, we control environmental factors and ensure that variation in microbial community composition and ecosystem functions in the microcosms are driven by dispersal.

Rainwater was collected at each site by using a spore trap constructed out of a sealed sterile 946 ml mason jar with a funnel inserted in the lid, following [30]. Rainwater gathered by spore traps was collected four times over the winter, at ~50-day intervals (mean = 48 days) starting in November. Four collections of spore traps over the course of the entire rainy season can make us collect more fungal and bacterial taxa, as different fungi produce spores at different times [30], and the interval between each collection ensured that sufficient rainwater had been collected in the spore traps. Before inoculating, rainwater was mixed well and filtered through sterile cheesecloth to remove debris.

The wood-soil microcosm consisted of 473 ml volume mason jars, each containing 60 g of locally collected soil (Tomales Bay, Point Reyes National Seashore, United States) and a wood block. Wood blocks were cut from *Pinus radiata*, the sister species of *P. muricata*, with uniform square 5 cm × 5 cm blocks, avoiding knots and irregularities (Pine Cone Lumber, Sunnyvale, CA, USA). Methods for wood block preparation were adapted from Fukami, Dickie [31]. In brief, blocks were oven dried for 48 h at 40 °C, weighed individually, soaked in deionized water for 48 h and then placed into the mason jars on top of soil. Microcosms were then autoclaved twice at 121 °C for 45 min, with a time gap of at least 24 h, as in Fukami, Dickie [31].

After preparation of rainwater and wood-soil microcosms, 3 ml rainwater of each site was randomly inoculated on woodblock in microcosms, with 10 replicates for each of the 17 sites. Ten control microcosms were inoculated with 3 ml sterile distilled water instead, for a total of 180 microcosms. Three subsequent inoculations were performed at ~48-day intervals, for a total of four inoculations. Microcosms were arranged in a randomized blocked design and incubated in the dark for one year in laboratory cabinets (mean ambient temperature of 21.8 °C; mean ambient humidity of 38.6%). The microcosms were sealed with jar lids. The jar lid had a hole with a filter in order to gas exchange without spores or conidia escaping. The location of experimental blocks in the laboratory was rotated monthly.

At the end of one-year incubation, each wood block was individually weighed field moist. Then, an ethanol-flamed drill bit was used to drill three holes lengthwise along each wood block in order to collect sawdust for DNA analysis, which was immediately frozen at −80 °C. After sawdust extraction, wood blocks were weighed, oven dried to a constant mass at 40 °C following Fukami, Dickie [31], and weighed again. We thereby determined field moisture levels and back-calculated the full dry weight of each wood block, allowing us to quantify total mass loss. Soil from each microcosm was divided into two parts, with one part frozen at −80 °C for DNA analysis, and another part used to measure soil carbon and nitrogen after lyophilization.

In field situation, soil is likely to be the major source of wood inhabiting fungi [37], and only a small fraction of airborne spores may succeed in colonization [50]. But this does not mean that our laboratory study system, only including airborne microbes in rainwaters, is completely different from in situ field situation. Actually, dispersal potential of bacteria and wood decay fungi is high [51, 52] and soil is also one of the major sources for airborne microbes [53], wood in our study system therefore can also capture some same microbes as field situation though the finer composition may be different. Thus, quantitative results of our study system can be helpful to understand in situ deadwood decomposition.

### Soil carbon and nitrogen

Soil total carbon and nitrogen were measured by a TOC analyzer (Shimadzu TOC-L). Soil water-soluble organic carbon and total nitrogen were extracted with deionized water (soil: water = 1: 10) by shaking for 30 min on a reciprocal shaker at 150 rpm. Supernatant was then filtered through a 0.45 μm polyether sulfone filter. Total organic carbon and total nitrogen in the filters were measured with a TOC analyzer (TOC-5000 Shimadzu), and were considered WSOC and WSTN, respectively. Water extractable organic matter is the most labile and mobile form of soil organic matter [54, 55], and is easily accessible to microorganisms, and therefore is sensitive to microbial activity. Percents of reduction of soil C and N were the shifts relative to the soil C and N of Control microcosms.

### Molecular methods and bioinformatics

Soil DNA was extracted by Qiagen DNeasy PowerSoil kit (Cat. No. 47014), according to manufacturer instructions. The fungal internal transcribed spacer (ITS) region was amplified with pair primers ITS1F-KYO1 (5’-CTHGGTCATTTAGAGGAASTAA-3’) and ITS2-KYO2 (5’-TAGAGGAAGTAAAAGTCGTAA-3’) [56], and the bacterial 16S rRNA gene was amplified with updated 515f (5’-GTGYCAGCMGCCGCGGTAA-3’) and 806R (5’-GGACTACNVGGGTWTCTAAT-3’) pair primers [57]. After amplifying and library preparation (*Supporting Information*), we submitted the library for 2 × 300 Illumina MiSeq sequencing to the Stanford Functional Genomics Facility.

We received a total of 11,216,759 demultiplexed reads for ITS amplicon and 9,589,246 demultiplexed reads for 16S rRNA gene amplicon across 170 samples, excluding experimental negative controls. We then used a DADA2 (1.12) workflow [58] to quality filter (ITS: maxN = 0, maxEE = c(2, 2), truncQ = 9, minLen = 50; 16S: truncLen = c(180,160), maxN = 0, maxEE = c(2,2), truncQ = 2), denoise, merge forward and reverse reads and remove chimeric sequences, leaving us with 7,555,208 reads for ITS amplicon and 6,877,195 reads for 16S rRNA gene amplicon. assignTaxonomy function of dada2 package (1.12) was used to taxonomic assignment. Fungal taxonomic assignment was done with the UNITE database [59], and bacterial taxonomic assignment was done with the Silva reference database [60, 61]. Before statistical analyses, we rarefied to 5000 reads per sample for fungal community analyses, and rarefied to 4500 reads per sample for bacterial community analyses, balancing sample retention and read depth. As for the ten controls samples, only two of the ten controls detected few fungal taxa, and bacterial community composition of these control samples were simple and were different from that of treatment samples (Supplementary Fig. S7).

### Statistical analysis

All analyses were performed using R statistical programming language version 4.0.3 [62]. Metabarcoding data were processed and analyzed using R package phyloseq [63]. Some blocks gained mass over the course of the experiment, presumably due to prolific fungal growth subsidized by nutrients and carbon from the underlying soil. These replicates were excluded from the primary analysis. Excluding these samples did not influence the statistical results much (Supplementary Table S1). In total, we retained 142 replicates for downstream analysis. Before statistical analysis, distance from forest edge was log10-transformed as in prior work at this study site [16, 30, 48, 64].

Kruskal-Walli’s test was used to compare soil C and N between treatment microcosms and control microcosms. Spearman’s Rank correlation coefficient was used to analyze the relationships among distance, fungal and bacterial alpha diversity and relative abundance, soil C and N, and wood mass loss. Fungal and bacterial community composition were visualized by plotting axis from non-metric multidimensional scaling (NMDS) analysis using metaMDS function of vegan package [65], with an envfit analysis from envfit function of vegan package [65] to analyze the relationship between distance and fungal and bacterial community composition.

To investigate the correlation between soil WSTN reduction, wood mass loss and microbial community at finer taxonomical level, redundancy analysis (RDA) of vegan package [65] was used to analyze the relationship between top 25 fungal genera or top 25 bacterial family and soil WSTN reduction and wood mass loss. We analyzed bacteria at the family level as there were a large part of unassigned bacterial genera. Fungal or bacterial Shannon diversity index was also included in the RDA to detect which phyla correlated with shifts of fungal or bacterial Shannon diversity.

## Supplementary information

Supplemental materials

## Data Availability

Raw sequences are available from the United States National Center for Biotechnology Information Sequence Read Archive (BioProject PRJNA952829). Data and scripts used are deposited in the figshare (10.6084/m9.figshare.22561042).
