# Supplementary material for: Dispersal changes soil bacterial interactions with fungal wood decomposition
Source: ISME Commun. 2023 May 3;3:44. doi: 10.1038/s43705-023-00253-5 (PMC10156657; doi:10.1038/s43705-023-00253-5)
Supplement: Supplementary file 1 — Supplemental materials [file 43705_2023_253_MOESM1_ESM.pdf]

Dispersal changes soil bacterial interactions with fungal wood decomposition

Cong Wang, Gabriel Reuben Smith, Cheng Gao, Kabir G. Peay

Kabir G. Peay
Email: kpeay@stanford.edu

This PDF file includes:

Supplementary text
Figures S1 to S9
Table S1

Supplementary Information Text

Material and Methods

Molecular methods and bioinformatics. The first polymerase chain reaction (PCR) for amplifying ITS or 16S rRNA genes consisted of 35 cycles of denaturation at 95°C, annealing at 50°C for ITS or 52.5°C for 16S rRNA gene, and extension at 72°C. Under the same temperature settings, a second PCR with eight cycles was used to attach molecular tags for high-throughput sequencing. After verifying by gel electrophoresis, the PCR products were cleaned with magnetic bead purification method using Sera-Mag SpeedBeads (MilliporeSigma, Munich, Germany; (1)). Then, we measured the DNA concentration in our cleaned PCR product using a Qubit fluorometer (Thermo Fisher, Waltham, MA, USA) and pooled our samples into a single library at equimolar concentration.

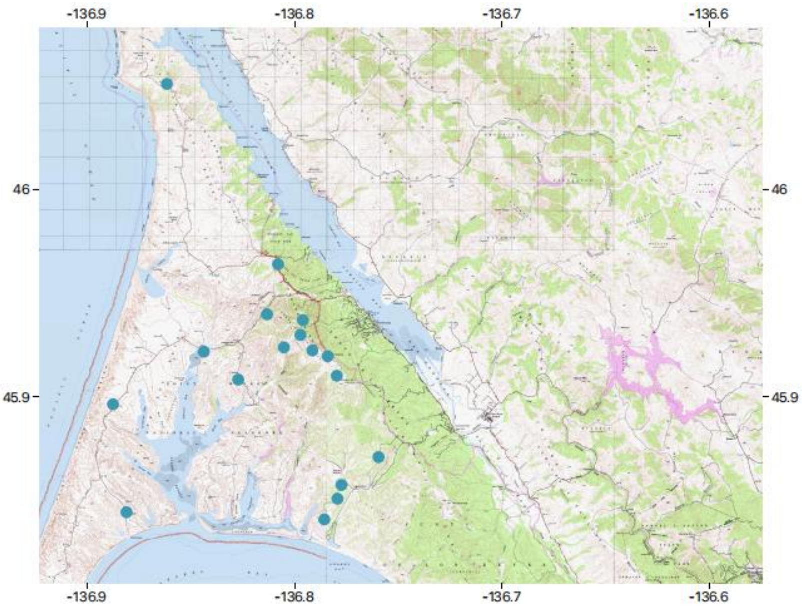

Fig. S1. Map of the seventeen sampling sites at Point Reyes National Seashore, California, United States (2).

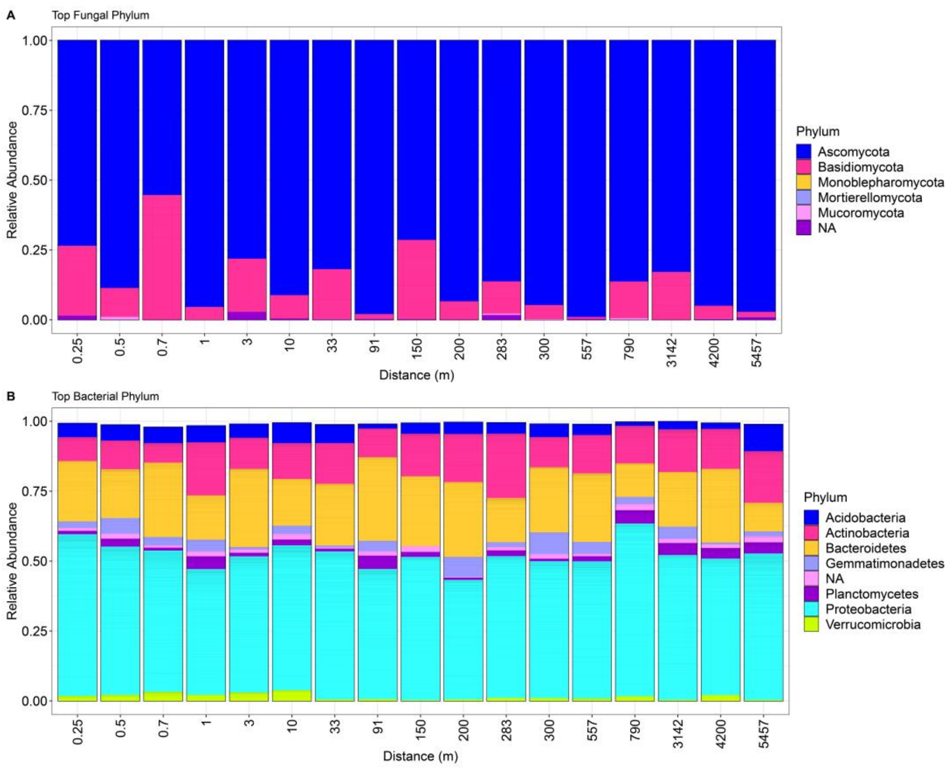

Fig. S2. The relative abundance of soil bacteria and fungi across the distance gradient (distance from the pine forest edge). (A) The relative abundance of fungal phylum. (B) The relative abundance of bacterial top eight phylum.

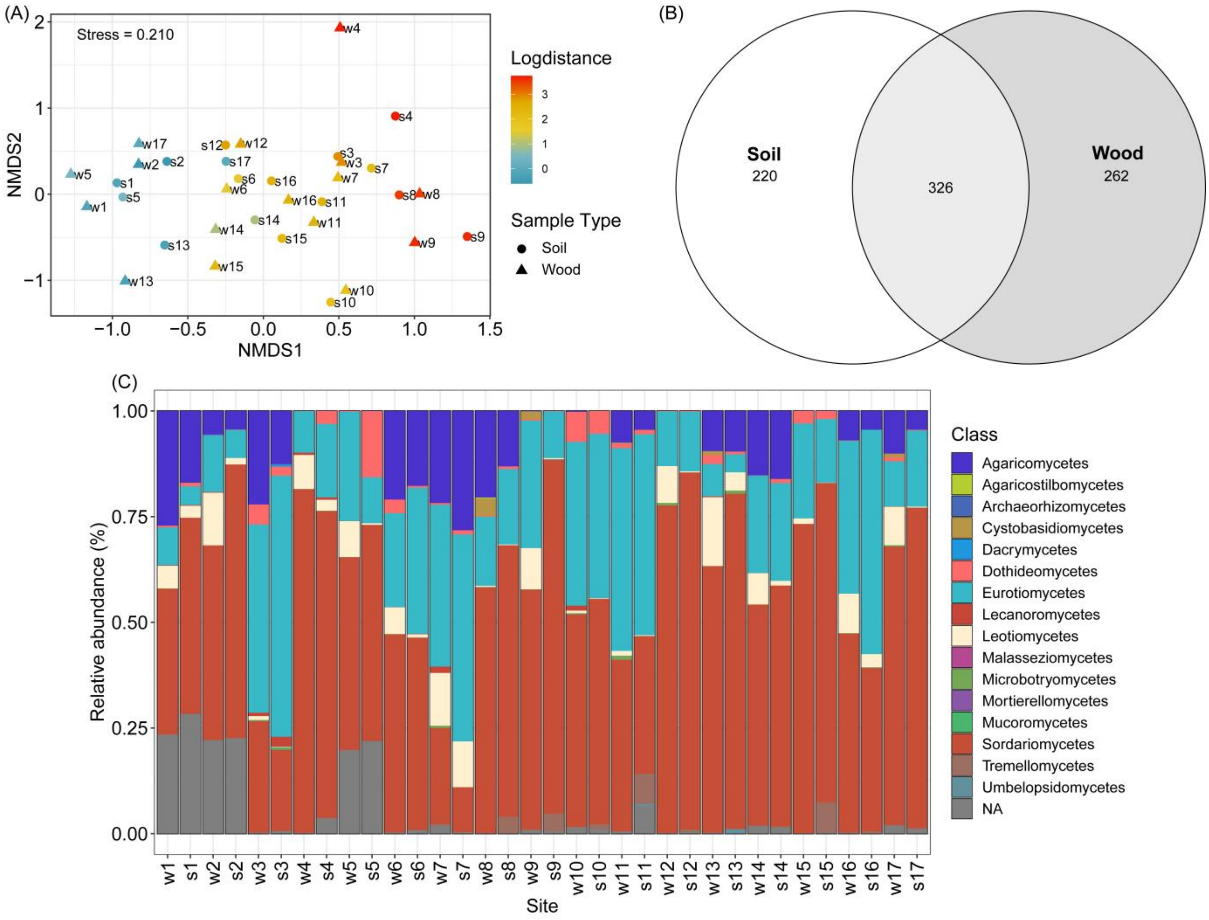

Fig. S3. Comparison of fungal community composition between wood and soil in experimental microcosms. (A) Non-metric multidimensional scaling (NMDS) analysis of wood and soil fungal community, coded by substrate and distance from forest. (B) Venn diagram of wood and soil fungal ASVs. Of the total 808 fungal ASVs across both wood and soil, shared ASVs account for ca. 60% of wood ASVs and 55% of soil ASVs. (C) The relative abundance of fungal classes in wood and soil. The lowercase letter and numeral represent substrate and site, respectively. For example, w1 and s1 represent wood and soil of site 1, respectively.

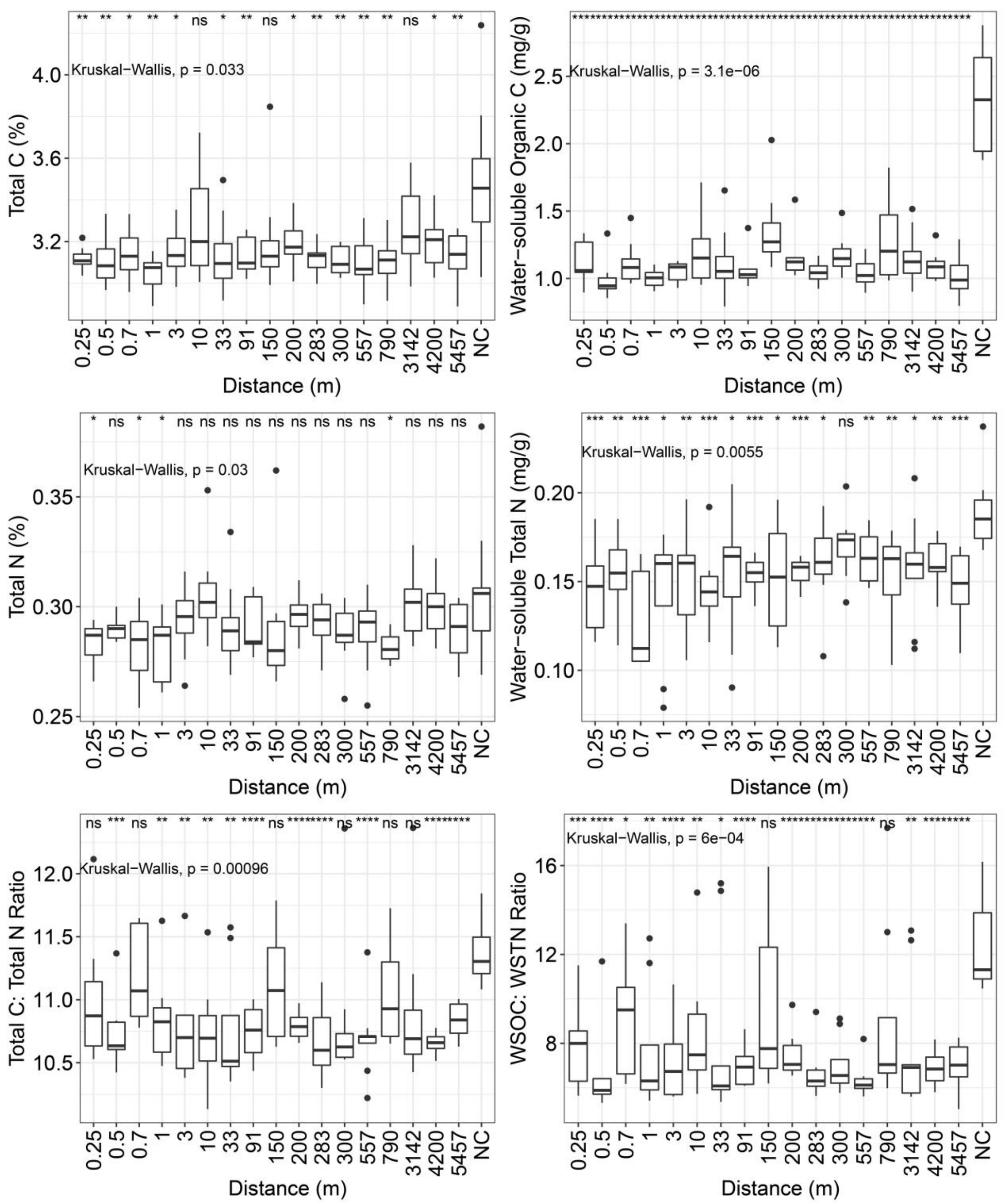

Fig. S4. Effects of rainwater addition on soil total C, total N, water-soluble organic C and water-soluble total N. Compared to the Controls, treatment with rain water decrease soil total C, water-soluble organic C and water-soluble total N. Decrease in soil total N is not as significant as total C and water-soluble total N. This is reasonable as microbes can respire more C, and microbes can assimilate water-soluble N as biomass while measurement of total N contain microbial biomass N. NC represent negative control treatments.

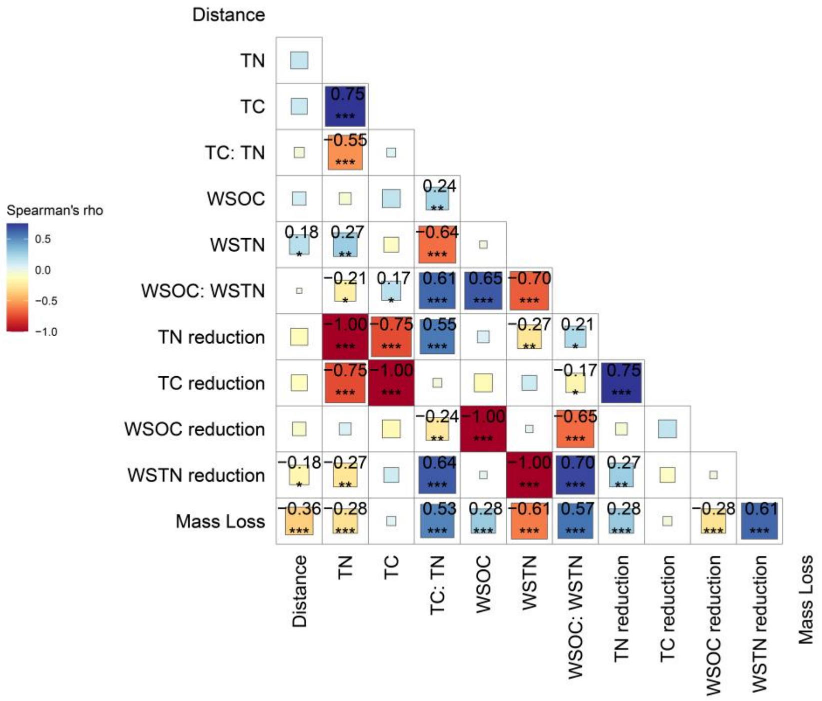

Fig. S5. Relationships among soil C, soil N, wood mass loss and distance from the edge of forest. TN: soil total N, TC: soil total C, WSOC: water-soluble organic C, WSTN: water-soluble total N. n = 142 for all analysis. * P < 0.05, ** P < 0.01, *** P < 0.001.

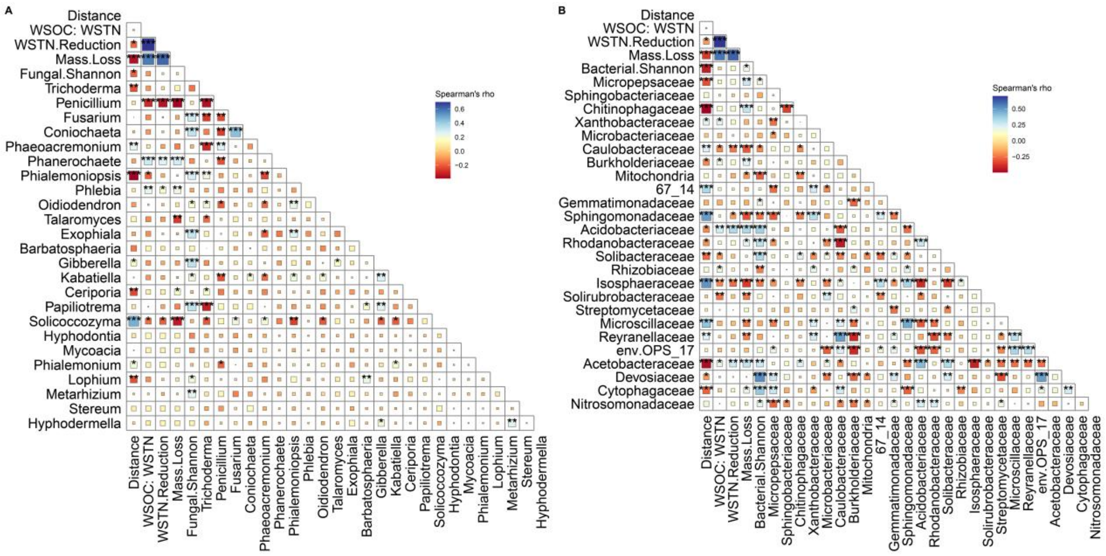

Fig. S6. Relationships among soil properties, wood mass loss and top fungal genera or top bacterial family across the distance gradient from the edge of forest. n = 142 for all analysis. WSOC: water-soluble organic C, WSTN: water-soluble total N. * P < 0.05, ** P < 0.01, *** P < 0.001.

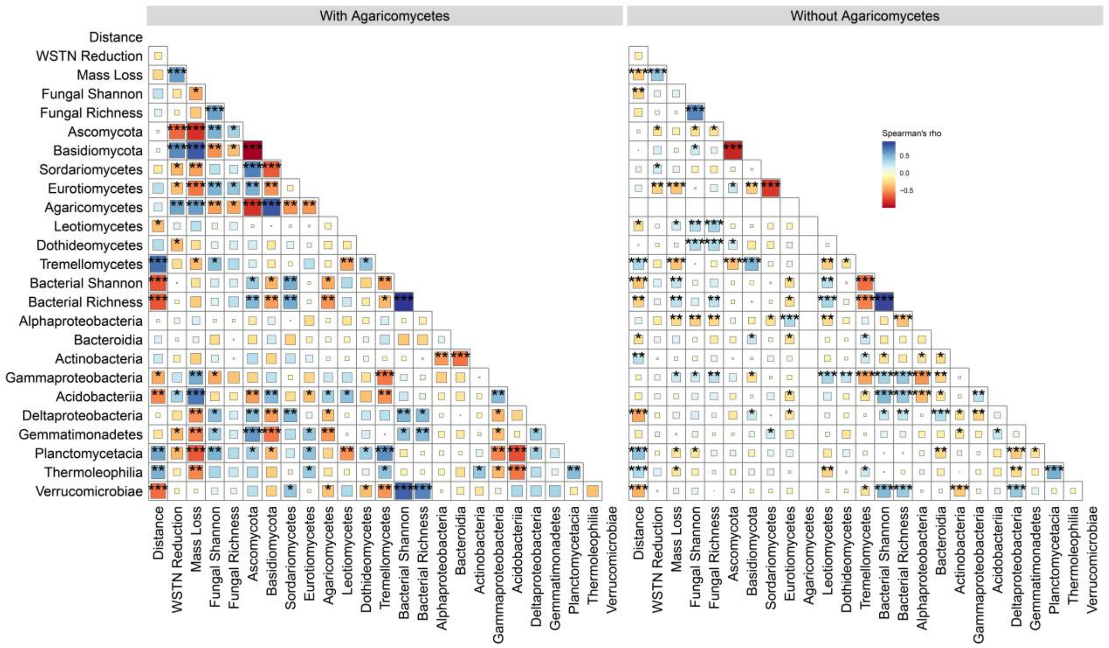

Fig. S7. Effect of the presence and absence of Agaricomycetes on the relationship between soil microbial community characteristics, wood mass loss percent and soil WSTN reduction percent. With Agaricomycetes: n = 31, without Agaricomycetes: n = 111, WSTN: water-soluble total N. * $P < 0.05$, ** $P < 0.01$, *** $P < 0.001$.

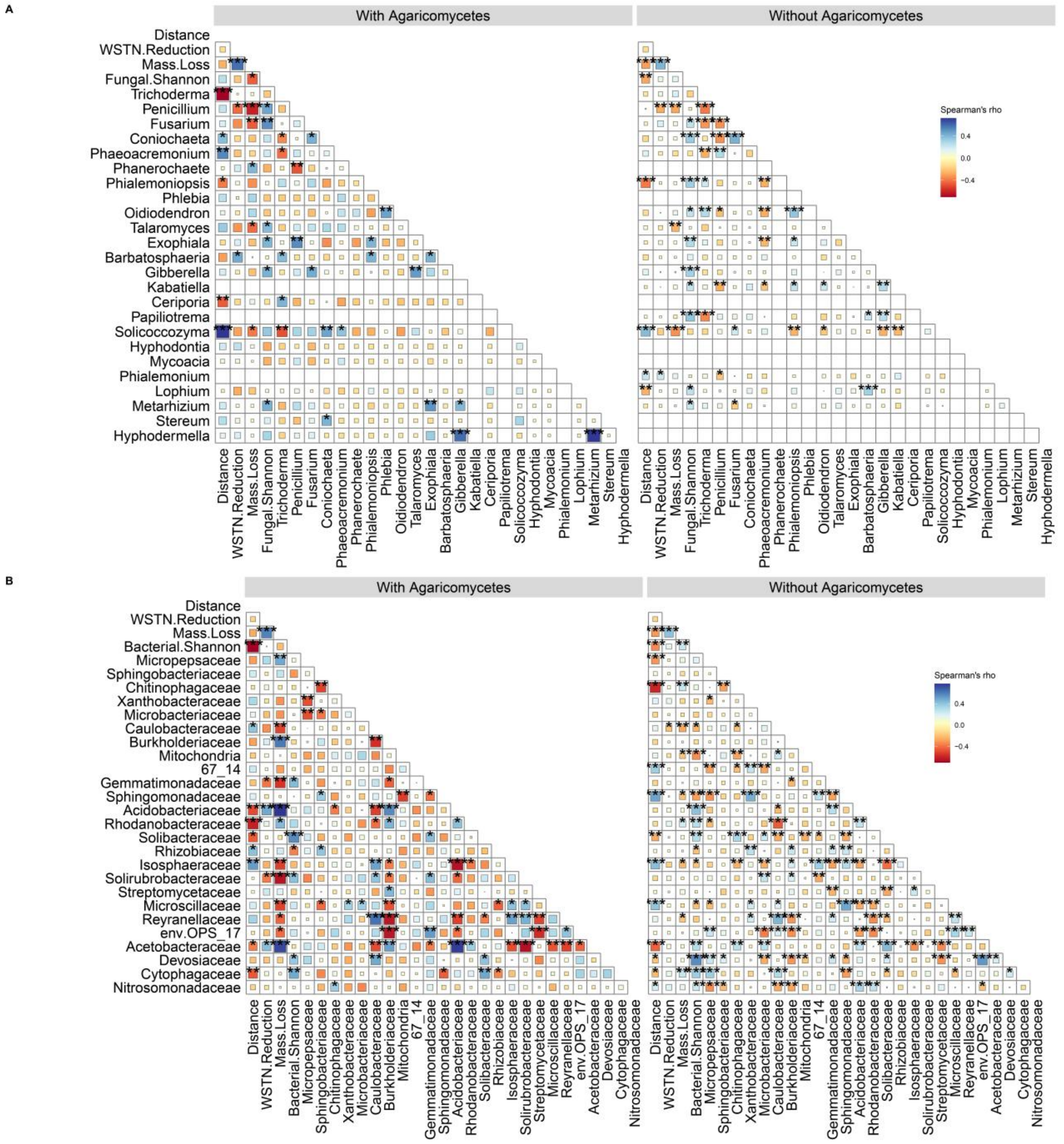

Fig. S8. Effect of the presence and absence of Agaricomycetes on the relationship among top fungal genera or bacterial families, wood mass loss percent and soil WSTN reduction percent. With Agaricomycetes: n = 31, without Agaricomycetes: n = 111, WSTN: water-soluble total N. * P < 0.05, ** P < 0.01, *** P < 0.001.

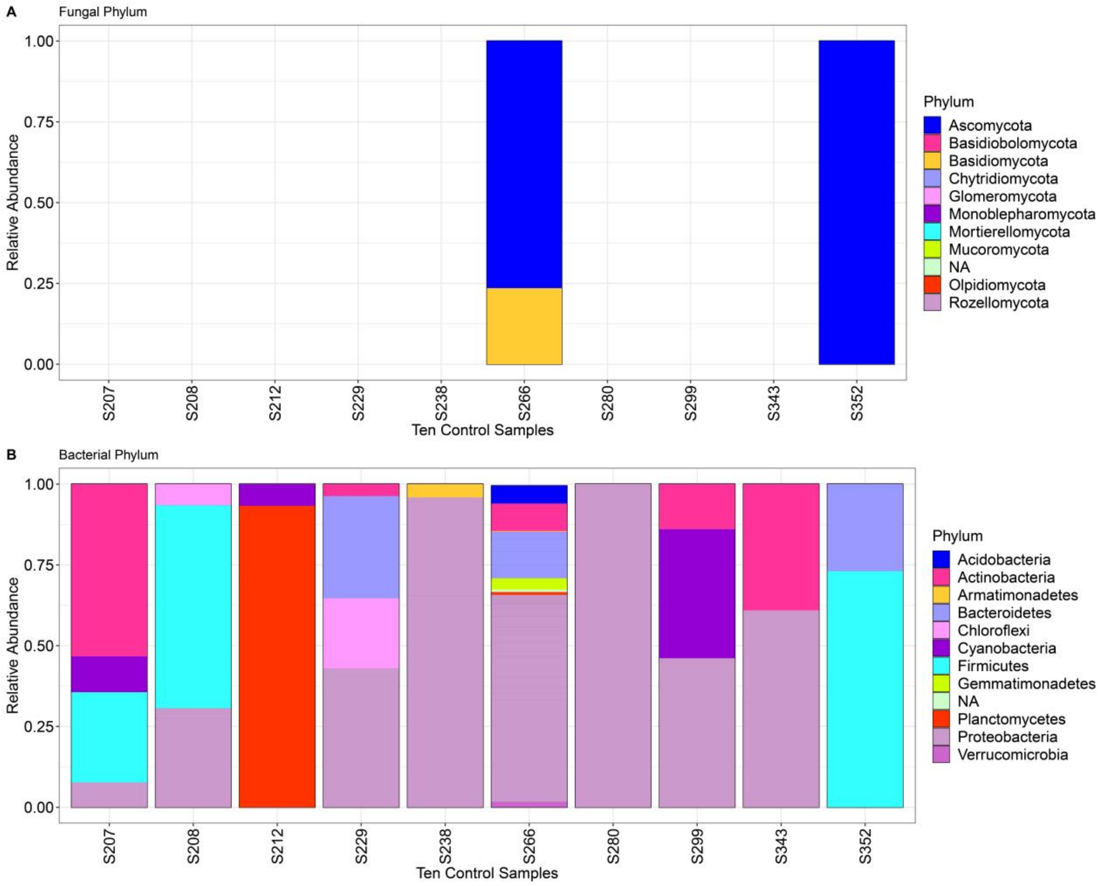

Fig. S9. Fungal and bacterial taxa detected in control sample of this study. Only two of the ten controls detected fungal taxa, and bacterial community composition of these control samples are simple and are different from that of treatment samples.

Table S1 Soil microbial community and ecosystem functions across the distance gradient from forest edge. * P < 0.05, ** P < 0.01, *** P < 0.001.

	Excluding negative mass loss (n = 142)		Including negative mass loss (n = 162 and 161 for bacteria)	
	Rho	PERMANOVA	Rho	PERMANOVA
Fungal richness	-0.08		-0.07	
Fungal Shannon	-0.19 *		-0.16 *	
Fungal community		R^2: 0.0817 ***		R^2: 0.0725 ***
Bacterial richness	-0.38 ***		-0.36 ***	
Bacterial Shannon	-0.43 ***		-0.42 ***	
Bacterial community		R^2: 0.0730 ***		R^2: 0.0702 ***
Mass loss	-0.36 ***		-0.30 ***	
WSTN reduction	-0.18 **		-0.22 **	
Mass loss ~ WSTN reduction	0.63 ***		0.59 ***	

Note: samples was deleted due to negative mass loss or sample reads rarefaction.

SI References

1. Talbot JM, Bruns TD, Taylor JW, Smith DP, Branco S, Glassman SI, et al. Endemism and functional convergence across the North American soil mycobiome. Proc Natl Acad Sci U S A. 2014;111(17):6341-6.
2. Smith GR, Peay KG. Multiple distinct, scale-dependent links between fungi and decomposition. Ecol Lett. 2021;24(7):1352-62.
